# Anticancer Potential and Other Pharmacological Properties of *Prunus armeniaca* L.: An Updated Overview

**DOI:** 10.3390/plants11141885

**Published:** 2022-07-20

**Authors:** Dusanka Kitic, Bojana Miladinovic, Milica Randjelovic, Agnieszka Szopa, Javad Sharifi-Rad, Daniela Calina, Veronique Seidel

**Affiliations:** 1 Department of Pharmacy, Faculty of Medicine, University of Niš, Ave. Zorana Djindjica 81, 18000 Nis, Serbia; dkiticyu@yahoo.co.uk (D.K.); bojana.miladinovic@medfak.ni.ac.rs (B.M.); milica.randjelovic@medfak.ni.ac.rs (M.R.); 2 Chair and Department of Pharmaceutical Botany, Jagiellonian University, Medical College, Medyczna 9, 30-688 Krakow, Poland; a.szopa@uj.edu.pl; 3 Facultad de Medicina, Universidad del Azuay, Cuenca 14-008, Ecuador; 4 Department of Clinical Pharmacy, University of Medicine and Pharmacy of Craiova, 200349 Craiova, Romania; 5 Natural Products Research Laboratory, Strathclyde Institute of Pharmacy and Biomedical Sciences, University of Strathclyde, Glasgow G1 1XQ, UK

**Keywords:** *Prunus armeniaca*, amygdalin, adjuvant therapy, anticancer mechanisms, toxicity

## Abstract

*Prunus armeniaca* L. (Rosaceae)-syn. *Amygdalus armeniaca* (L.) Dumort., *Armeniaca armeniaca* (L.) Huth, *Armeniaca vulgaris* Lam is commonly known as the apricot tree. The plant is thought to originate from the northern, north-western, and north-eastern provinces of China, although some data show that it may also come from Korea or Japan. The apricot fruit is used medicinally to treat a variety of ailments, including use as an antipyretic, antiseptic, anti-inflammatory, emetic, and ophthalmic remedy. The Chinese and Korean pharmacopeias describe the apricot seed as an herbal medicinal product. Various parts of the apricot plant are used worldwide for their anticancer properties, either as a primary remedy in traditional medicine or as a complementary or alternative medicine. The purpose of this review was to provide comprehensive and up-to-date information on ethnobotanical data, bioactive phytochemicals, anticancer potential, pharmacological applications, and toxicology of the genus *Prunus armeniaca*, thus providing new perspectives on future research directions. Included data were obtained from online databases such as PubMed/Medline, Google Scholar, Science direct, and Wiley Online Library. Multiple anticancer mechanisms have been identified in in vitro and in vivo studies, the most important mechanisms being apoptosis, antiproliferation, and cytotoxicity. The anticancer properties are probably mediated by the contained bioactive compounds, which can activate various anticancer mechanisms and signaling pathways such as tumor suppressor proteins that reduce the proliferation of tumor cells. Other pharmacological properties resulting from the analysis of experimental studies include neuroprotective, cardioprotective, antioxidant, immunostimulatory, antihyperlipidemic, antibacterial, and antifungal effects. In addition, data were provided on the toxicity of amygdalin, a compound found in apricot kernel seeds, which limits the long-term use of complementary/alternative products derived from *P. armeniaca*. This updated review showed that bioactive compounds derived from *P. armeniaca* are promising compounds for future research due to their important pharmacological properties, especially anticancer. A detailed analysis of the chemical structure of these compounds and their cytotoxicity should be carried out in future research. In addition, translational pharmacological studies are required for the correct determination of pharmacologically active doses in humans.

## 1. Introduction

Various parts of *Prunus armeniaca* Lam., commonly known as the apricot tree, are used medicinally to treat a wide range of diseases, including respiratory, gynecological, and digestive disorders and for their antipyretic, anti-inflammatory, hepatoprotective, vulnerary, anthelmintic, and anticancer properties [1]. Apricot fruits are rich in dietary fibres, proteins, sugars, fatty acids, micronutrients, volatile compounds, carotenoids, phenolics, and lignans. Apricot kernels contain cyanogenic glycosides, with the main constituent (up to 4.9%) identified as amygdalin [2]. Cyanogenic glycosides are found in particularly high amounts in bitter apricot varieties. Most pharmacological investigations carried out to date have focused on apricot fruits and kernels [3]. The biological effects reported have included protective activity on the heart, antioxidative/radical scavenging, neuroprotective, anti-hyperlipidemic, hepatoprotective, antimicrobial, antiparasitic, antiviral, anti-inflammatory, analgesic, immunomodulatory, and anticancer activity [4]. All apricot parts exhibit various pharmacological effects. Most of the studies that have been carried out so far have reported the pharmacological investigation of apricot fruits and seeds. Various parts of the apricot plant are used worldwide as a complementary and alternative medicine (CAM) to treat cancer, and some preliminary clinical studies have already indicated a promising potential for apricot-based products in this field [5]. The present work aimed to provide up-to-date knowledge on botany, traditional uses, phytochemistry, pharmacological properties, and the potential use of *P. armeniaca* L. in the treatment of cancer.

## 2. Review Methodology

This review aimed to identify the anticancer and other beneficial pharmacological effects of *P. armeniaca* on human health. The literature and published papers reporting experimental studies (including molecular mechanisms) on cells (in vitro) and animal (in vivo) models, as well as clinical studies, which focused on the cytotoxic, anticancer, cancer chemo-preventive, and other beneficial pharmacological effects of *P. armeniaca* were retrieved and critically analysed. The following MeSH terms were used for the search “*Prunus armeniaca*/chemistry”, “Plant extracts/chemistry”, “Plant extracts/pharmacology”, “Amygdalin/pharmacology”, “Seeds/chemistry”, “Antineoplastic agents/pharmacology”, “Antineoplastic agents/therapeutic use”, “Humans”, “Neoplasms/drug therapy”, “Antineoplastic agents”, “Phytogenic/pharmacology”, “Apoptosis/drug effects”, “Cell cycle checkpoints/drug effects”, “Cell proliferation/drug effects”, “Drug screening assays”, “Antitumor”, “Humans”, “Mice”. Information on the plant taxonomy and the chemical structures was validated using World Flora Online and PubChem, respectively [6].

## 3. Botany

*P. armeniaca* is thought to originate from the northern, north-western, and north-eastern provinces of China, although some data show that it may also come from Korea or Japan. The cultivation of this species then spread to Central Asia, Armenia, and Anatolia, before being imported by the Romans to European countries such as Italy and Greece. Apricots were brought to North America by English and Spanish travellers [2,7]. Today, it is grown extensively in Europe, Asia, and America [8,9]. The plant is a small to medium-sized deciduous tree with a height of approximately 4 m under cultivation conditions, but it can reach up to 10–15 m in height in its natural habitat. It is characterized by a reddish grey-brown bark, with young reddish leaves and twigs. The leaves are alternately arranged, simple, oval to round-oval (5–12 × 5–10 cm), with sharp pointed tips and irregularly serrated edges. They have a smooth surface when mature, with dark red stalks 2–4 cm long, bearing glands [9,10]. Flowers develop in early March to early April. They are white to pinkish (2–3 cm), solitary, with five red sepals, opening before the leaves. The corolla of the flower consists of five orbicular, oval, or obovate petals with pink veins. There are many erect stamens with yellow anthers and the ovary is in a perigynous position. The fruit is a drupe (3, 5–8 cm), often asymmetrical, in a globose, ovoid, obovoid, or amygdaloid shape with a fleshy outer layer surrounding a stony flattened, smooth endocarp. The skin color varies from yellow to reddish with or without bloom, light pubescent or semi-glabrous. The seeds are bitter or sweet, flat, cordate, with a thick yellowish to deep brown skin and dark brown veins radiating upwards (1.1–1.9 cm × 0.8–1.5 cm × 0.4–0.8 cm thick). They have a short linear hilum at the sharp end and a chalaza at the rounded end [9,10].

## 4. Traditional and Ethnomedicinal Importance

The dried ripe seeds of apricots (*Semen Armeniacae*) have been reported as a plant material of special medicinal interest. They are used to treat gynecological diseases, rheumatic pain, headache, and skin hyperpigmentation, with the seed oil employed for skin diseases, ear inflammation, and tinnitus [11]. A decoction is used to treat asthma, productive cough, and fever while the seed oil is used for constipation [11,12].

Apricots are ingredients of two traditional Chinese medicines—Jinhuaqinggan granules and Lianhuaqinwen capsule/granules—which are used in combination with conventional medicines in the treatment of viral infections [13]. In Korea, the seeds are used to treat cough, phlegm and the common cold. In European countries, they are used as an aphrodisiac. In Vietnam, they are used to treat respiratory and digestive disorders, while in India it is used as an antidiarrhoeic, antipyretic, emetic, hepatoprotective, and anthelmintic agent [8,14]. In Algeria, apricot leaves, flower, seed, and fruit are used for cancers, prostate enlargement, and pyelonephritis [15]. In India, the ground seeds are used for skin rashes as a paste made with water [16]. The seeds, rich in amygdalin, are also used for their analgesic, spasmolytic, anthelmintic, anti-asthmatic, antitussive, expectorant, demulcent, emollient, pectoral, sedative, laxative, and vulnerary properties. Amygdalin has been used, in very small amounts, for preventing and treating cough, asthma, constipation, migraine, and hypertension, as well as for cancer treatment in Russia [17]. Apricot flowers are claimed to promote fertility in women, while the roots are used for coughs, bronchitis, asthma, and obstipation and to soothe inflamed or irritated skin [8,14]. The seeds, and the oil they produce, are used to treat vaginal infections, tumors, ulcers, anorexia, and disturbed sleep. Combined with peach and walnut seeds, they are used for upper respiratory tract infections, bronchitis, asthma, and pulmonary tuberculosis. They are also used in cosmetic products [17].

The fruit, seed, stem, and gum of the apricot are used as an anticancer remedy in Turkey, Pakistan, China, India, and Western Caucasus [18,19]. The Turks use fresh apricot fruits and seeds for intestinal cancers [20]. In Morocco, apricot leaves, fruits, and seeds are used as a decoction, an oil, or in a powdered form as an anticancer remedy [21]. In Traditional Chinese Medicine (TCM), bitter apricot seeds are one of the most commonly used remedies to improve respiratory function in patients with non-small cell lung cancer [22,23]. The seed is also included in several preparations as a keratolytic agent in the escharotic treatment of skin cancer [24]. In a study conducted in Turkey, apricot fruits were identified as the most commonly used CAM treatment to alleviate chemotherapy-induced constipation in cancer patients [25]. Preparations containing apricot seeds are in the top five Chinese herbal products commonly used in Taiwan [26]. Products such as Qing-Zao-Jiu-Fei-Tang/Ma-Xing-Shi-Gan-Tang and Ma-zi-ren-wan have been demonstrated to reduce the mortality risk of patients with lung cancer and cervical cancer, respectively [27,28]. Apricot seeds are also used as a CAM for breast cancer patients in Malaysia [29].

## 5. Chemistry and Bioactive Compounds

The apricot fruit is rich in proteins, monosaccharides, polysaccharides, fats, acids, and dietary fibres. It also contains micronutrients such as vitamins (A, B group, C, E, and K), minerals, amino acids, and fatty acids, contributing to its significant nutritive value [30]. Several volatile compounds contributing to the aroma of the fruit have been reported, with the major compounds identified as *β*-ionone, linalool, *γ*-decalactone, hexanal, (*E*)-2-hexenal, (*E*,*E*)-2,4-decadienal, (*E*)-2-nonenal, *γ*-dodecalactone [31], carotenoids (*β*-carotene, *β*-cryptoxanthin, *γ*-carotene, lycopene), and phenolic compounds (chlorogenic, neochlorogenic, caffeic, galllic, ferrulic and *p*-coumaric acid, (+)-catechin, (−)-epicatechin, proanthocyanidins, cyanidins, kaempferol, and quercetin glycosides) [30,32].

Dried apricot fruits contain lignans such as secoisolariciresinol with phytoestrogen-like properties [33]. Apricot kernels contain cyanogenic glycosides. These compounds are considered antinutritional, and their aglycones produce the toxic compound hydrocyanic acid (HCN) [34,35]. Some cultivars, known as bitter apricots, contain high amounts of cyanogenic compounds, equivalent to 240–350 mg of hydrocyanic acid or hydrogen cyanide (HCN) per 100 g, whilst sweet apricots contain almost none [34,36]. The amygdalin content in the seeds can vary between 3–4 and 8% [37].

**Figure 1:**
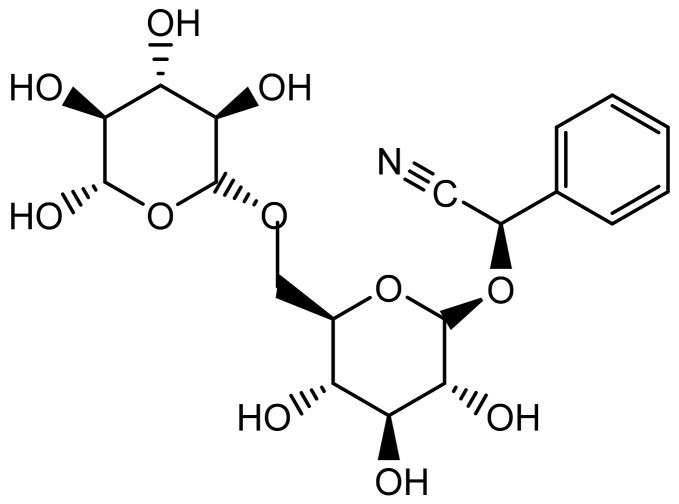
Chemical structure of amygdalin.

The major constituent of apricot seeds (up to 4.9%) is the cyanogenic glycoside amygdalin (Figure 1), followed by other cyanogenic compounds such as prunasin and mandelonitrile. Other constituents include various fatty acids, mostly oleic, palmitic and linoleic acid, phytosterols, essential amino acids, the enzyme emulsin, as well as vitamins and minerals [8,11,38,39]. Amygdalin, sometimes referred to as vitamin B17, although it is not classified as a vitamin, has a molecular formula of C_20_H_27_NO_11_. It contains benzaldehyde, hydrocyanic acid, and two β (1–>6) linked d-glucose units (gentiobiose) [40,41,42]. Amygdalin is hydrolyzed by emulsion after tissue rupture, giving gentiobiose and l-mandelonitrile. Gentiobiose is further hydrolyzed to afford glucose while mandelonitrile is degraded to yield benzaldehyde and HCN [43].

## 6. Anticancer Activities: Underlying Multi-Targets Mechanisms

Cancerous tumors occur when cells divide rapidly and begin to invade nearby tissues and spread to other areas of the body (metastases) [44,45,46]. The main goals of cancer treatment are to cure the disease, as well as prolong and improve the quality of a patient’s life [47,48]. The effectiveness of treatment depends on the correct administration, early detection, accurate diagnosis of cancer and compliance with standards of care [49,50]. Chemotherapy has severe side effects; therefore, natural remedies based on bioactive cytotoxic compounds are a valuable aid in cancer prophylaxis, but also a complementary alternative medicine (CAM) to various forms of cancer [51,52,53]. Cancer patients in the UK reported the use of apricot-derived products in CAM [54,55]. In Australia, a small proportion of patients from a healthcare unit (5%) reported the use of apricot kernels as an alternative remedy for breast or colon cancer [56]. Natural bioactive compounds can strengthen the immune system and have an antitumor effect, either directly or by inhibiting angiogenesis, preventing the proliferation of tumor cells or metastases [57,58,59,60].

Preclinical pharmacological studies have shown the potential mechanisms of apricot anticancer activities by using aqueous and/or alcoholic/hydroalcoholic extracts of *P. armeniaca* L. Aqueous extracts are prepared from different parts of the plant, using water as a solvent, preferably distilled or softened, because water is very well tolerated by tissues. Water is the most common solvent used in pharmacy and for obtaining plant protection products. However, only water-soluble active substances are dissolved in water at a pH close to neutral (acids, bases, salts, sugars, phenols and polyphenols, amino acids, glycosides, gums, tannins, enzymes), but they do not dissolve resins, alkaloids, oils, fats, and hydrocarbons; therefore, in some cases, it is recommended to slightly alkalize the water with baking soda or acidify it with citric acid [61]. Alcoholic/hydroalcoholic extracts are prepared from different parts of the plant or plant mixtures, using as a solvent ethanol/methanol of different concentrations [62]. Alcohol has a good ability to dissolve organic and mineral substances, dissolving, to a greater or lesser extent: salicylic acid, volatile oils, dyes, lecithin, balms, or resins [61]. Different alcohol concentrations are recommended for the preparation of extractive alcoholic solutions [63,64]. Although it has several advantages (it evaporates easily, is a good antiseptic, inhibits enzyme activity, does not influence hydrolysis, precipitates albuminoid materials, and can serve to remove them), the use of alcohol as a solvent also has disadvantages: the odor of extractive solutions does not have the same power of penetration through cellular membranes, such as water. This is why the better extractive solution from different species is the hydroalcoholic one [65].

**Figure 2:**
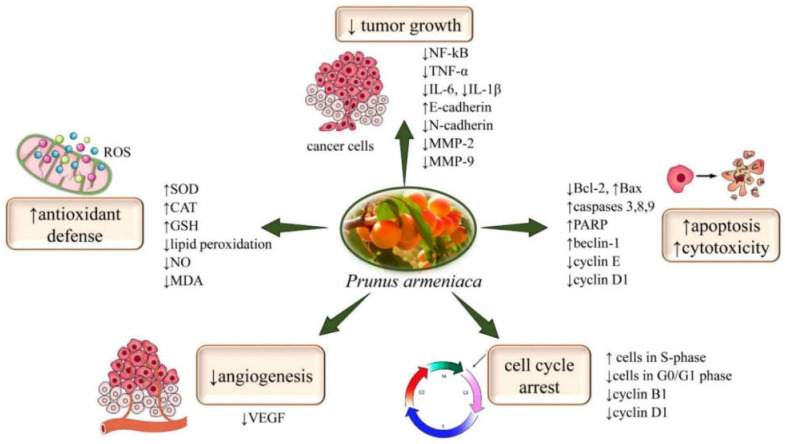
Diagram showing the potential molecular anticancer mechanisms of *P. armeniaca*. Its bioactive compounds displayed anticancer activity by ↑apoptosis, ↑cytotoxicity, ↓angiogenesis, and cell cycle arrest. In addition, *P. armeniaca* biocompounds have a dual beneficial effect on oxidative stress in cancer; they stimulate antioxidant defense by increasing antioxidant markers such as SOD, CAT, and GSH, and by decreasing the levels of pro-inflammatory cytokines such us NF-κB, TNF α, interleukins in tumor mass, it has an antioxidant effect, thus reducing the growth of cancer cells. Abbreviations and symbols: ↑increase, ↓decrease, nuclear factor kappa B (NF-κB), tumor necrosis factor α (TNF α), interleukin (IL), matrix metalloproteinase (MMP), superoxide dismutase (SOD), catalase (CAT), glutathione (GSH), malondialdehyde (MDA), nitric oxide (NO), poly-ADP ribose polymerase (PARP), VEGF (vascular endothelial growth factor).

The most representative mechanisms involved in the anticancer activity of *P. armeniaca* L. are summarized in Figure 2 and Table 1.

### 6.1. Cancers of the Nervous System

The aqueous extract of apricot seeds induced apoptotic neuronal cell death in mouse N2a neuroblastoma cells. Treatment with the extract increased the expression of the pro-apoptotic protein Bax and the activity of the caspase-3 enzyme while decreasing the expression of the anti-apoptotic protein Blc2 [66]. The ethanolic extract of the seeds was characterized as a weak tumoricidal agent on the same cells with LC_50_ > 5.0 mg/mL [67]. The antiproliferative properties of apricot pulp after freezing, canning, or drying were investigated on rat C6 glioma cells. The effects observed were dose-dependent, with the highest activity reported for the methanolic extract. Canned apricots showed the strongest antiproliferative effect inhibition, followed by dried, and then frozen apricots. It is considered that the process of canning releases bioactive compounds that are responsible for the antiproliferative effects. The drying and freezing process also affects phytochemicals, though to a lesser extent, and can degrade phenolic compounds and carotenoids, resulting in neo-formed antiproliferative compounds [68].

### 6.2. Digestive Cancers

#### 6.2.1. Oral Cancer

Ethanolic apricot seed extract showed activity on KB oral cancer cells. The maximum activity (82%) was achieved at a concentration of 100 µg/mL (IC_50_ value of 61 µg/mL) [69]. Another study demonstrated the inhibitory effect of apricots—along with carrot, burdock, and prune—extracts in the lipid peroxide-induced 8-hydroxydeoxyguanosine (8-OH-dG) formation in vitro. Significant inhibition of the 8-OH-Dg formation was identified for extracts and fractions containing chlorogenic acid. The latter was also able to inhibit 8-OH-dG formation in 4-nitroquinoline-1-oxide-induced carcinogenesis in the tongues of rats [70].

#### 6.2.2. Gastric Cancer

An ethanolic apricot flesh extract showed activity (58%) towards AGS human gastric carcinoma cells at the concentration of 4 mg/mL [71].

#### 6.2.3. Liver Cancer

An aqueous-methanolic apricot seed extract and its amygdalin-containing fraction displayed anticancer activity by inducing apoptosis and autophagy, reducing cell proliferation, increasing antioxidant defenses, and reducing the release of the pro-inflammatory marker TNF-α and the vascular endothelial growth factor (VEGF)—a marker of angiogenesis. The antiproliferative activity on HepG2 cells was evidenced with IC_50_ values of 25.26 µg/mL and 6.20 µg/mL after 24 h and 48 h, respectively [72]. A trypan blue exclusion test showed cytotoxicity on Ehrlich ascites carcinoma (EAC) cells with IC_50_ values of 20.2 µg/mL for the extract and 7.8 µg/mL for the amygdalin-containing fraction, respectively. A hydroethanolic extract prepared from apricot seeds, with octasiloxane-hexadecamethyl as the main component, showed a cytotoxic effect against HepG-2 cells with an IC_50_ value of 22.8 μg/mL. The antiproliferative activity of this extract was investigated on mice inoculated with EAC cells. Treatment with the extract (100 mg/kg) for 6 days showed a significant decrease in tumor volume and cell count compared to the control. In addition, the extract improved the liver and kidney functions, as estimated by the levels of AST, ALT, urea, creatinine, MDA, and SOD and CAT activity [69]. Aqueous, methanolic, and ethanolic extracts from apricot kernels significantly inhibited the growth of hepatocellular HCT-116 cells in a concentration-dependent manner (IC_50_ values of 17.5, 19.2, and 14.5 µg/mL, respectively) [73].

A 20% ethanolic extract prepared from kernels of bitter apricots of Bulgarian origin, containing the cyanogenic glycosides amygdalin, deidaclin, linamarin, and prulaurasin, demonstrated antigenotoxic, antirecombinogenic, antimutagenic, and anticarcinogenic effects in yeast cell-based assays. When tested on HepG2 cells, the extract showed a cytotoxic effect (23% and 32% cell viability at 2.5 and 5 μg/mL, respectively). At 5 μg/mL, the extract showed antiproliferative activity with an IC_50_ value of 3.77 ± 0.45 μg/mL [74]. Extracts prepared from kernels of 19 cultivars of apricot were tested for antiproliferative activity toward HepG2 cells. The best activity was observed for the Waflu Chuli apricot cultivar (EC_50_ of 14.71 ± 0.82 mg/mL) [72]. An ethanolic extract from apricot flesh showed strong cytotoxicity (91.9%) at a concentration of 4 mg/mL on Hep3B hepatocellular carcinoma cells [71].

The apricot seed extract and its amygdalin-containing fraction also showed anticancer activity in vivo in a 7,12-dimethylbenz[a]anthracene (DMBA)-induced carcinogenesis mice model. They protected the liver against oxidative damage by reducing lipid peroxidation and increasing the antioxidant response measured via SOD, CAT, GSH, and MDA levels. The activity of the amygdalin-containing fraction was linked to the presence of amygdalin. The latter was able to convert to HCN in tumor tissue and induce oxidative-dependent apoptotic cell death. Treatment with the apricot seed extract and its amygdalin-containing fraction also increased the mRNA expression of caspase-3 (by 120% and 244%, respectively) and Beclin-1 (by 128% and 186%, respectively). It also decreased the gene expression of Bcl-2 by −37% and −73%, respectively [75].

Another study showed that an apricot fruit diet supplementation provided high protection against the oxidative stress induced by radiotherapy and DMBA in the liver of rats. An apricot fruit-supplemented diet (20%) in DMBA- and radiotherapy-treated animals reduced oxidative stress and significantly decreased ALT, AST, 5′NT, MDA, and NO levels and the expression of Bcl-2, activator protein 1 (AP-1), cAMP response element-binding protein (CREB), and NF-κB, while significantly increasing Bax, caspase-3, and GSH activity. Histopathological examinations revealed that mitosis, pericentral necrosis, and pleomorphism caused by DMBA were mitigated after apricot and/or radiotherapy administration [76].

Ethanolic extracts (70% and 99.9%) prepared from apricot seeds also showed activity in *N*-nitrosodiethylamine-induced hepatocellular carcinogenesis in rats. The extracts, both in a dose of 200 mg/mL and after 8 weeks of treatment, significantly reduced the elevated levels of AST, ALT, ALP, total and direct bilirubin, albumin, total proteins, alpha-fetoprotein (tumor marker), MDA, and NO, and they increased the reduced glutathione levels in liver tissue. Similar effects were found for animals that received amygdalin or silymarin (50 mg/kg) [77].

Mice fed with raw or heat-processed apricot kernels and transplanted with EAC cells showed a strong reduction in tumor growth. The administration of heat-processed apricot kernels also prolonged the animal lifespan compared to the control group [78].

#### 6.2.4. Colon Cancer

The aqueous, methanolic, and ethanolic extracts from apricot kernels dose-dependently inhibited the growth of HCT-116 colon cells with IC_50_ values of 33.6 and 36.3 µg/mL, respectively [73]. The growth of HCT-116 colon cells increased from 79.0 ± 1.5% to 90.6 ± 4.6% after treatment with a fermented methanolic apricot seed extract at the concentration of 100 µg/mL [79].

Extracts (80% ethanol) prepared from apricot kernels of South African and Chinese origin showed a significant impact on the cell proliferation, apoptosis, and cell cycle progression of HT-29 colon cancer cells. The South African extract had a bi-phasic proliferative effect after 24 h, stimulating cell proliferation at the lowest and highest concentrations (100 and 1000 μg/mL) but inhibiting it at the middle concentration of 500 μg/mL. After 72 h, the low concentrations inhibited cell proliferation, while cell proliferation increased with extracts at 500 μg/mL. The Chinese extract decreased proliferation after 24 h and 48 h in a dose-dependent manner. Changes in morphology were noticed for cells treated with the Chinese kernel extracts after 24 h and the South African kernel extract at 1000 μg/mL after 72 h (irregularly shaped cells/cellular shrinking) [80].

A fruit beverage (consisting of apricot, orange, and grape) subjected to in vitro gastrointestinal digestion showed high antiproliferative activity against CaCo-2 human colon cancer cells. The latter, upon continuous incubation with this digest, had their cell cycle interrupted in the S-phase, associated with reduced cyclin B1 and D1 levels [81]. In a similar study, a digested beverage fortified with zinc and milk showed high activity after 24 h of incubation against Caco-2 and HT-29 cells (35% and 29% inhibition of proliferation, respectively). The mechanism of action was correlated with an increase in the proportion of cells in the S-phase and a decrease in the number of cells in G0/G1, but no difference in the number of cells in the G2/M phase compared to the control [82].

Along with its antigenotoxic, antirecombinogenic, antimutagenic, and anticarcinogenic effects demonstrated in yeast cell-based assays, a 20% ethanolic extract prepared from kernels of bitter apricots of Bulgarian origin, containing amygdalin, deidaclin, linamarin, and prulaurasin, showed cytotoxicity against HT-29 cells (32% and 41% cell viability at 2.5 and 5 μg/mL, respectively). At a concentration of 5 μg/mL, the extract caused a weak antiproliferative effect (IC_50_ > 5 μg/mL) [74]. Another study showed that apricot extracts could decrease P-glycoprotein-mediated efflux mechanisms in Caco-2 cells [83].

A diet containing 20% of sun-dried and sulfur-fumigated apricot (SDA and SFA) fruits reduced oxidative stress and telomerase activity in azoxymethane-induced carcinogenesis in rats. Telomerase is a ribonucleoprotein complex that is important for the maintenance of telomeres length and cellular immortality. Telomerase activity was significantly reduced in animals fed apricots compared to the control group (from 54.25 to 23.54 RTA (relative telomerase activity)/g proteins for SFA and 3.42 for SDA). SDA was more effective in suppressing telomerase activity, while SFA had better antioxidative activity, increasing GSH levels and decreasing NO and MDA levels [84].

#### 6.2.5. Pancreatic Cancer

The ethanolic extracts of bitter and sweet apricot kernels, as well as amygdalin, inhibited the growth of PANC-1 human pancreatic cancer cells in a time- and dose-dependent manner (IC_50_ values of 704, 945, and 35 µg/mL after 72 h, respectively) without a significant effect on 293/KDR normal epithelial cells. The bitter apricot kernel extract was more effective than the sweet apricot kernel extract. DAPI staining and flow cytometry identified fragmented and condensed nuclei, as well as an increased number of early and late-stage apoptotic cells, respectively. Apoptosis in the PANC-1 cells was confirmed by the upregulation of Bax and caspase-3 and the downregulation of Bcl-2 gene expression, evaluated by real-time PCR. The results indicated that these extracts, as well as amygdalin, induced apoptosis in pancreatic cancer cells through a mitochondrial-dependent pathway [85,86].

### 6.3. Breast Cancer

An extract from apricots significantly inhibited the proliferation of MCF-7, HDF, and MDA-MB-231 human breast cancer cells in a concentration-dependent manner after 24, 48, and 72 h. The IC_50_ values of this extract against MCF7, HDF, and MDA-MB-231 cells after 72 h were 0.5, 1.51, and 0.48 mg/mL, respectively. The expression levels of Bax and c-FLIP regulatory genes, obtained from the total RNA of MCF-7 and MDA-MB-231 cells, were reduced in the presence of the extract in a time-dependent manner [87].

The aqueous, methanolic, and ethanolic extracts from apricot kernels inhibited the growth of MCF-7 cells in a dose-dependent manner (IC_50_ values of 38.9, 34.9, and 33.9 µg/mL, respectively) [73]. Furthermore, a hydroethanolic apricot seed extract also showed antiproliferative activity against MCF-7 cells with an IC_50_ value of 31.5 μg/mL. The main component in the extract was detected by GC-MS and identified as octasiloxane-hexadecamethyl [69]. The aqueous, ethyl acetate, and hydromethanolic extracts of apricot seeds showed antiproliferative activity in MCF-7, MDA-MB-231, and T47D breast cancer cells. The hydromethanolic extract showed better activity in all cell lines after 48 h of incubation (IC_50_ values of 0.198, 0.693, and 0.532 mg/mL, respectively). This extract increased the number of cells in G0/G1 and decreased the number of cells in the G2/M phase. Apoptosis was induced via an increase in the pro-apoptotic proteins, Bax and caspase-3, and a decrease in the anti-apoptotic protein Blc2 [88].

Soltani et al. [89] investigated the extracts of four Iranian apricot cultivars, Jahangiri, Palmia, Jafari, and N585. Hydroethanolic extracts prepared from apricot seeds were evaluated for their antiproliferative effect on MCF-7 cells at 25, 100, 400, and 1200 μg/mL. The strongest activity was achieved by the N585 cultivar after 24 h and 72 h at the highest concentration of 1200 μg/mL [89].

An ethanolic extract of apricot flesh showed cytotoxicity (72.8%) on MCF-7 cells at a concentration of 4 mg/mL [71]. A multifruit polyphenolic preparation containing apricot, peach, chokeberry, raspberry, wild strawberry, bilberry, and cranberry was evaluated for its cytotoxicity on T47D human breast ductal cancer, MCF-7 breast adenocarcinoma, and MCF-12A normal breast cells. This polyphenolic preparation showed concentration-dependent cytotoxicity towards MCF-7 and T47D cancer cells (IC_50_ = 1.2 μg/mL) and MCF-12A cells (IC_50_ = 0.6 μg/mL). Microscopic investigations confirmed the cytopathogenic effect of this preparation on all three cell lines. The preparation was less cytotoxic on cancer cells than on normal cells, which could be explained by the interference of berry polyphenols with estrogen receptors leading to modifications in the production of paracrine growth factors [90]. An ethyl acetate apricot leaf extract reduced the survival rate of MCF-7 cells, promoting apoptosis and increasing the levels of reactive oxygen species. The extract up-regulated Bax, down-regulated Bcl-2, reduced the expression of CDK4, cyclin E, and cyclin D1, and increased caspase-3 activity [91].

Salarbashi et al. [92] investigated the cytotoxicity of curcumin encapsulated with apricot gum exudate and compared it with pure curcumin. The authors reported that both samples were toxic to 4T1 breast cancer cells in a concentration-dependent manner, with curcumin encapsulated nanoparticles showing a stronger effect, most likely as a result of the synergism with the apricot gum exudate [92].

### 6.4. Lung Cancer

An ethanolic extract of apricot flesh showed strong cytotoxicity (88.2% at a concentration of 4 mg/mL) on A549 human lung carcinoma cells [71]. Fei-Liu-Ping (FLP) is an oral TCM used to treat lung cancer and contains apricot, among other medicinal plants. FLP was reported to inhibit the growth of A549 cells through the regulation of NF-κB and by changing the expression of E-cadherin, N-cadherin, and matrix metalloproteinase-2 (MMP-2) and matrix metalloproteinase-9 (MMP-9). In addition, FLP significantly reduced tumor growth by 40% in Lewis lung-xenografted mice, and its combination with cyclophosphamide reduced tumor growth by 83.23%. It was observed that the serum levels of pro-inflammatory cytokines IL-6, TNF-α, and IL-1β were decreased, while some improvement was found in the expression of E-cadherin, the inhibition of N-cadherin, and matrix metalloproteinase-9 [93].

In a study conducted in a Malaysian hospital, about 30% of cancer patients receiving chemotherapy used apricot seeds [94]. A clinical study demonstrated that a decoction called Bufei Huayu containing bitter apricot seeds and other plants and used in TCM, in combination with gefitinib, had a significant effect in the treatment of advanced non-small-cell lung cancer. This treatment showed good safety, improved patients’ prognosis, and reduced the risk of thrombosis. In addition, this herbal preparation significantly reduced some of the adverse effects of gefitinib, such as skin rash or elevated ALT levels [95].

### 6.5. Urogenital Cancer

An apricot seed extract and its hexane, ethyl acetate, and water fractions inhibited 12-*O*-tetradecanoylphorbol-13-acetate-induced ornithine decarboxylase activity in T24 human bladder carcinoma cells (IC_50_ values > 20 µg/mL) [96]. The aqueous extract of bitter apricot seeds induced apoptosis in DU145 human prostate cancer cells via caspase-3 activation, the up-regulation of Bax expression, and the down-regulation of Bcl-2 expression [97].

An ethanolic apricot flesh extract displayed potent cytotoxicity (89.4%), at a concentration of 4 mg/mL, on human cervical adenocarcinoma (HeLa) cells [71]. Salarbashi et al. [92] investigated the cytotoxicity of curcumin encapsulated with apricot gum exudate and compared it with pure curcumin. The authors reported that curcumin encapsulated with apricot gum exudate inhibited the growth of A2780 human ovarian cancer cells in a concentration-dependent manner, with curcumin encapsulated nanoparticles showing a stronger effect as a result of the synergism with the apricot gum exudate [92].

### 6.6. Skin Cancer

Apricot seed essential oil inhibited the growth of HaCaT cells (IC_50_ value of 142.45 μg/mL). The study of the mechanism of action showed G0/G1 cell cycle arrest, increased numbers of early and late-stage apoptotic cells, the activation of caspases-3/8/9, Bax, and PARP, and a decrease in Bcl2 and Rel/NF-κB levels. Apoptosis was mediated through the death receptor, mitochondrial, and NF-κB pathways [98]. The apricot fruit extract (95% ethanolic) had an inhibitory effect on 12-O-tetradecanoylphorbol-13-acetate-induced Epstein-Barr virus early antigen (EBV-EA) activation in vitro, which has been strongly correlated with the inhibition of skin carcinogenesis in mice [99].

### 6.7. Leukemia

Aqueous, ethyl acetate, and hydromethanolic extracts from apricot seeds inhibited the growth of NALM-6 and KG-1 acute leukemia cells without toxicity on normal control cells. The ethyl acetate extract, containing 0.67% of amygdalin, showed the strongest activity (IC_50_ values of 0.388 mg/mL and 0.159 mg/mL on NALM-6 and KG-1 cells, respectively). Flow cytometric analyses revealed that this extract, at the IC_50_ concentrations, caused almost 50% apoptosis in both cell lines after 48 h. A significant increase in caspase-3 gene expression was observed in both cell lines [100]. Another study showed that mice fed with apricot kernels (2 g), two days before the transplantation of LYO-1 lymphosarcoma cells and for one month, showed a significant reduction in tumor growth compared to the control group [101].

**Table 1:** Anticancer preclinical studies and potential mechanisms of action of natural compounds from *Prunus armeniaca*.

Cancer Type	Model	Main Cellular Effects	Ref
Cancers of the nervous system	N2a neuroblastoma cells in vitro	↑Bax, ↑caspase-3, ↓Blc2 LC_50_ > 5.0 mg/mL	[66]
	C6 glioma cells in vitro	antiproliferative effect	[68]
Digestive cancers	KB oral cancer cells in vitro	↓8-OH-dG IC_50_ = 61 µg/mL	[69]
	AGS human gastric carcinoma cells in vitro	↓cell proliferation IC_50_ = 4 mg/mL	[71]
	HepG2 cells in vitro	↑apoptosis, ↑autophagy, ↑antioxidant defenses antiproliferative, ↓angiogenesis ↓TNF-α, ↓VEGF IC_50_ = 25.26 − 6.20 µg/mL	[72]
	HCT-116 cells in vitro	IC_50_ = 17.5, 19.2, 14.5 µg/mL	[73]
	mice inoculated with EAC cells in vivo	↓tumor volume, ↓AST, ↓ALT, ↓urea, ↓creatinine, ↓MDA, ↓SOD, ↓CAT Dose = 100 mg/kg	[69]
	HepG2 cells in vitro	↑cytotoxic effect	[74]
	HepG2 cells in vitro	antiproliferative EC_50_ = 14.72 ± 0.82 mg/mL	[71]
	DMBA-induced carcinogenesis mice in vivo	antioxidant, ↓lipid peroxidation, ↓SOD, ↓CAT, ↓GSH, ↓MDA ↑caspase-3, ↑Beclin-1, ↓Bcl-2	[75]
	*N*-nitrosodiethylamine-induced hepatocellular carcinogenesis in rats in vivo	↓AST, ↓ALT, ↓ALP, ↓bilirubin, ↓alpha-fetoprotein, ↓MDA, ↓NO, ↓glutathione Dose = 200 mg/mL	[77]
	transplanted EAC cells in mice in vivo	↓tumor growth	[78]
	HCT-116 colon cancer cells in vitro	↓cancer cell growth IC_50_ = 33.6 − 36.3 µg/mL	[73]
	HCT-116 colon cancer cells in vitro	↓cancer cell growth IC_50_ = 100 µg/mL	[79]
	HT-29 colon cancer cells in vitro	↓cell proliferation	[80]
	Caco-2 human colon cancer cells in vitro	cell cycle interrupted in the S-phase, ↓cyclin B1 ↓D1 levels	[81]
	Caco-2 and HT-29 cells in vitro	↓proliferation ↓cells in G0/G1	[82]
	HT-29 cells in vitro	↑cytotoxicity IC_50_ = 2.5 − 5 μg/mL antiproliferative IC_50_ > 5 μg/mL	[83]
	PANC-1 human pancreatic cancer cells in vitro	↓growth, ↑apoptosis, ↑Bax, ↑caspase-3, ↓Bcl-2 IC_50_ = 704, 945, 35 µg/mL	[85,86]
Breast cancer	MCF-7, HDF, MDA-MB-231 human breast cancer cells in vitro	↓cell proliferation IC_50_ = 0.5, 1.51, 0.48 mg/mL	[87]
	MCF-7 cells in vitro	↓cell growth IC_50_ = 8.9, 34.9, 33.9 µg/mL	[73]
		IC_50_ = 31.5 μg/mL	[69]
	MCF-7, MDA-MB-231, T47D breast cancer cells in vitro	antiproliferative, ↑apoptosis, ↑Bax, ↑caspase-3, ↓Blc2, ↑cells in G0/G1 phase, ↑cells in the G2/M phase IC_50_ = 0.198, 0.693, 0.532 mg/mL	[88]
	MCF-7 cells in vitro	antiproliferative IC_50_ = 25, 100, 400, 1200 μg/mL	[89]
		↑cytotoxicity IC_50_ = 4 mg/mL	[71]
		↑apoptosis, ↑ROS, ↑Bax, ↑Bcl-2, ↓CDK4, ↓cyclin E, ↓ cyclin D1, ↑caspase-3	[91]
	T47D human breast ductal cancer, MCF-7 breast adenocarcinoma, MCF-12A normal breast cells in vitro	↑cytotoxicity IC_50_ = 1.2 μg/mL against MCF-7, T47D cells IC_50_ = 0.6 μg/mL against MCF-12A cells	[90]
Lung cancer	A549 human lung carcinoma cells in vitro	↑cytotoxicity IC_50_ = 4 mg/mL	[71]
		↑cytotoxicity, ↓NF-κB, ↓E-cadherin, ↓N-cadherin, ↓MMP-2, ↓MMP-9, ↓IL-6, ↓TNF-α, ↓IL-1β	[93]
Urogenital cancers	T24 human bladder carcinoma cells in vitro	antiproliferative ↑apoptosis IC_50_ > 20 µg/mL	[96]
	DU145 human prostate cancer cells in vitro	↑apoptosis, ↑Bax, ↑caspase-3, ↓Blc2	[97]
	HeLa human cervical adenocarcinoma cells in vitro	↑cytotoxicity, ↓cell growth IC_50_ = 4 mg/mL	[71]
Skin cancer	HaCaT cells in vitro	↓ cell growth, ↑caspases-3/8/9, ↑Bax, ↑PARP, ↓Bcl2, ↓NF-κB ↑G0/G1 cell cycle arrest IC_50_ = 142.45 μg/mL	[98]
Leukemia	NALM-6, KG-1 acute leukemia cells in vitro	↑apoptosis, ↑caspase-3 IC_50_ = 0.388 − 0.159 mg/mL	[100]

Symbols: ↑ increase, ↓ decrease.

## 7. Other Pharmacological Properties

### 7.1. Neuroprotective Activity

The methanol extract of apricot bark VEGF showed anti-acetylcholinesterase (AChE) activity [102]. The essential oil from apricot leaves inhibited acetylcholinesterase as well as butyrylcholinesterase, suggesting its potential for Alzheimer’s disease (AD) [103]. Apricot kernel extracts—especially the aqueous extract from bitter apricot kernels—demonstrated anti-AChE and neuroprotective activity [103]. Carotenoids, particularly lutein, showed potent anti-amyloidogenic activity in vitro, suggesting their potential benefit in AD [104].

### 7.2. Cardioprotective Activity

An apricot-rich diet (10% and 20% of the total diet) showed a significant impact on myocardial ischemia-reperfusion injury in rats. This cardioprotective effect was based on the reduction of infarct sizes, histopathological modifications, and change in antioxidant enzyme activities of the heart tissue [105]. Similar effects were observed with the oil from apricot kernels, which significantly improved the lipid status in rats [105,106].

Various peptides, obtained following the enzymatic hydrolysis of apricot kernel protein, inhibited the angiotensin-converting enzyme in vitro, indicating their potential antihypertensive activity [107].

### 7.3. Hepatoprotective Activity

The apricot and its preparations have been reported to reduce liver damage. The consumption of apricot seeds had an impact on liver microstructures in rabbits [108,109]. A dietary intake of apricot fruits has been demonstrated to reduce the extent of liver damage and steatosis caused by carbon tetrachloride (CCl_4_) in rats. In animals fed with apricots, serious damage to hepatocytes, edematous cytoplasmic matrix, large lipid globules, and degenerated organelles was significantly reduced. Oxidative stress, estimated by measuring MDA and glutathione levels as well as SOD, CAT, and GST-Px activity, decreased in comparison to CCl_4_-exposed rats [110]. Oral administration of a 70% and a 99.9% ethanolic extract of apricot seeds at a dose of 100 mg/kg, and of amygdalin and silymarin (50 mg/kg each), showed hepatoprotective activity, after CCl_4_ exposure, with a significant decrease in ALT (alanine aminotransferase), AST (aspartate aminotransferase), and ALP (alkaline phosphatase) levels and a significant increase in albumin and total proteins in rat sera [77]. Administration of a 3% and 5% bitter apricot kernel-containing food also significantly decreased CCl4-induced liver injury in rats by reducing AST, ALT, and hepatic Bcl-2 and NF-κB levels and increasing hepatic Bax, caspase-3, and Nrf2 levels. Such hepatoprotective effects were also observed during histopathological examinations [111]. The administration of apricot kernels to rats, at doses of 3 mg/kg and 6 mg/kg, significantly elevated serum levels of AST, GSH, and GPx and levels of GSH, SOD, and CAT in the hepatic tissue after 30 days. Histopathological examinations indicated hepatocyte enlargement with a dose of 3 mg/kg and hepatocyte hypertrophy, infiltration, and congestion with 6 mg/kg [112].

Another study demonstrated that the administration of dried apricot fruits and kernels to rats reduced ethanol-induced hepatotoxicity, with dried apricots decreasing the elevated levels of AST, ALT, and LDH and dried apricots and apricot kernels normalizing MDA concentrations in tissues [113]. Hepatoprotective effects against paracetamol-induced liver damage were also observed following the administration of sun-dried organic apricot fruit and leaf extracts to rats. Biochemical and histopathological analyses confirmed this protective activity [114,115].

Another study revealed that an extract prepared from apricot seeds prevented cyclophosphamide-induced hepatorenal damage and leukopenia in mice [116]. Dried apricot fruit also prevented acrylamide-induced liver and intestine damage in rats by decreasing plasma malondialdehyde (MDA) levels and improving antioxidant enzyme activities [117,118].

### 7.4. Metabolic Effects: Anti-Hyperlipidemic Activity

Ethanolic extracts from apricot fruits and petroleum ether extract from apricot seeds were characterized as anticholilithiatic agents in vitro, as they reduce the weight of cholesterol gallstones and increase levels of cholesterol in human bile [119]. A study in rats demonstrated that detoxified (i.e., devoid of HCN and other antinutritional components such as tannins, oxalates, and phytic acid) apricot kernel flour significantly increased HDL and decreased total cholesterol, LDL, and VLDL levels compared to a control (untreated) group [35]. A microencapsulated apricot kernel powder has been reported to significantly improve parameters related to diabetes and obesity in normal- and cafeteria-diet-fed rats. The powder increased the native thiol, total thiol, thioredoxin reductase, HDL levels, and antioxidant status while reducing the levels of disulfide, total cholesterol, LDL, triglyceride, glucose, catalase (CAT), superoxide dismutase (SOD), tumor necrosis factor-*α* (TNF-*α*), and glutathione peroxidase (GSH-Px), particularly in cafeteria-diet-fed rats [120]. In addition, apricot fruits can bind to bile acids in vitro, supporting their cholesterol-lowering activity [120].

The consumption of apricots has been linked with an anti-hyperlipidemic effect. Healthy volunteers consuming a diet comprised of apricot fruits (200 g daily over three weeks) experienced a moderate reduction in their triglycerides, total cholesterol, LDL, VLDL, and a significant increase in their HDL plasma levels [121]. Another study showed that the consumption of apricot seeds for 12 weeks reduced total cholesterol and LDL, but not HDL and triglycerides levels in healthy volunteers [122]. In women of reproductive age, the daily consumption of bitter apricot seeds has been reported to alter the endocrine and lipid profile. After 42 days, the level of LDL cholesterol reduced, while the levels of the follicle-stimulating hormone (FSH), testosterone, and androstenedione significantly increased [123].

### 7.5. Immunomodulatory Activity

Apricot kernel oil has been reported to significantly stimulate the immune system of cyclophosphamide-treated rats. Lymphocytes isolated from rats treated with the oil showed a significant increase in immunoglobulin (Ig)A, IgM, IgG, interleukin (IL)-2, IL-12, and TNF-α levels. The oil also reduced cyclophosphamide-associated oxidative stress and organ degeneration [124].

### 7.6. Antioxidant Activity

Oxidative stress is triggered by an imbalance between the amount of reactive oxygen species/free radicals, which cause harmful effects, and the body’s natural antioxidant defense mechanisms [125,126]. In recent decades, research has focused on finding natural antioxidant substances that can neutralize the negative potential of free radicals [127,128,129]. Previous studies have demonstrated the antioxidant and radical scavenging activity of the fruit (flesh), kernel, oil, pomace, bark, and leaf extracts in vitro [73,102,130]. It was noted that the radical scavenging activity of the fruit reduced following drying at high temperatures [131]. A flour prepared from peeled defatted kernels roasted showed high antiradical and reducing power, while an unroasted sample showed the best antilipoperoxidant activity [132]. Essential oil from apricot leaves (mostly containing phytol, manoyl oxide, linalool, limonene, and (*E*)-2-hexenal) also displayed excellent antiradical and anti-lipoperoxidant effects. Dried apricot fruits significantly prevented nephrotoxicity and intestinal oxidative damage caused by methotrexate, and they increased the levels of CAT, SOD, and glutathione, while decreasing the formation of malondialdehyde (MDA) in rats’ kidneys and intestines [133,134].

Apricot fruits have also been reported to alleviate the reactive oxygen species-related harmful effects of low-dose radiation on testis tissue in rats, before and after exposure. The histopathologic examination of rat tissues showed that a diet containing 20% of apricots significantly improved testicular oxidative status and altered TBARS (thiobarbituric acid reactive substances), SOD, CAT, and GSH-Px levels [135]. The protective antioxidant effects of apricot fruits have also been noticed on alcohol-induced testicular damage and radiation-induced kidney damage in rats. Thus, a diet rich in apricots significantly reduced histopathological changes in kidneys, including hemorrhage, interstitial fibrosis, glomerular collapse, and inflammatory infiltrates [136].

### 7.7. Anti-Inflammatory Activity

An extract from apricot kernels reduced prostaglandin E_2_ and NO levels in lipopolysaccharide (LPS)-stimulated murine BV2 microglial cells by downregulating the mRNA expression of cyclooxygenase-2 and inducible NO-synthase, respectively [137]. In addition, an apricot seed extract inhibited the specific binding of the pro-inflammatory mediator leukotriene B4 to human peripheral neutrophils [138]. An apricot kernel ethanolic extract and its combination with apricot oil significantly mitigated trinitrobenzene sulfonic acid induced-ulcerative colitis in rats. Macroscopic and microscopic changes were evaluated by calculating the ulcer and total colitis indices [139]. Apricot seed extracts mitigated corneal ulcers in pigeons and rabbits and keratoconjunctivitis sicca in rats through the inhibition of inflammation and matrix metalloproteinases [140,141,142].

### 7.8. Antimicrobial, Antiparasitic, Antiviral Activity

Synthetic antibiotics play an important role in the fight against bacterial infections, but they also have adverse effects and can increase bacterial resistance [143,144]. Natural antibiotics derived from plants with antibacterial properties are effective in combatting many types of bacteria that cause infections [125,145].

The apricot fruit, seed, leaf, root, and stem extracts have demonstrated antimicrobial activity in vitro against Gram-(+)/Gram-(−) bacteria and fungi [146,147,148,149]. Apricot extracts also inhibit the growth of *Mycobacterium tuberculosis* and *Helicobacter pylori* [148,150]. An ethanol extract of apricot seeds inhibited the mutagenicity of 4-nitroquinoline-1-oxide- and *N*-methyl-*N*-nitro-*N*-nitrosoguanidine in an Ames test using *Salmonella typhimurium* TA98 and TA100 strains [71], while an *n*-hexane extract inhibited the mutagenicity of 3-amino-1,4-dimethyl-5H-pyrido [4,3-b]indole,2-(2-furyl)-3-(5-nitro-2-furyl)acrylamide and benzo [*α*] pyrene in the same type of assay [151]. A leaf extract has revealed antiparasitic activity against *Leishmania tropica*, inhibiting the growth of promastigotes and amastigotes [152]. Seed extracts showed antiviral activity against A/H_1_N_1_ influenza and an inhibitory effect on HIV-1 protease.

### 7.9. Phytoestrogen-like Properties

Interest in phytoestrogen-like bioactive compounds has grown in recent years, as alternative treatments for menopause that have fewer side effects than synthetic estrogens are needed, as well as due to epidemiological evidence showing that females who traditionally consume phytoestrogen-containing plants have a lower incidence of osteoporosis [62,153,154]. Studies have revealed that bitter apricot seeds could affect plasma levels of the follicle-stimulating hormone (FSH) in rabbits, suggesting that their components may be involved in ovarian folliculogenesis [155]. Dried apricots (25% of the diet for eight weeks) have been reported to improve body and spine bone mineral density in osteopenic ovariectomized mice [156].

## 8. *P. armeniaca* Toxicity

Amygdalin found in *Prunus armeniaca* seeds is toxic (LD_50_ of 9279.5 mg/kg in rats), but its oral intake does not necessarily cause serious toxicity [34,157]. The range of critical concentrations from 0.5 to 3.5 mg/kg is only achieved by a massive and rapid intake. Excessive exposure to cyanogenic glycosides and HCN may lead to nausea, vomiting, diarrhea, dizziness, weakness, mental confusion, convulsions, coma, and eventually death. Hydrogen cyanide depresses cellular respiration by blocking mitochondrial electron transport and preventing oxygen uptake. The human body has the potential to detoxify cyanides through thiosulfate sulfur-transferase, which converts them into thiocyanates that are excreted in the urine [34,158]. Nevertheless, cyanide toxicity caused by the ingestion of apricot kernels has been reported [159,160,161,162]. This includes a report on a woman who had ingested about 15 g of apricot kernels, which resulted in a classical presentation of coma, decreased body temperature, and metabolic acidosis. Immediate improvement of the symptoms was only achieved after inhalation of amyl nitrite, followed by the intravenous administration of sodium nitrite and sodium thiosulfate [162]. The proteins in apricot seeds can cause allergies by reacting and binding to IgE [163].

## 9. Conclusions and Future Perspectives

The apricot plant, *P. armeniaca* L., exhibits a wide range of biological effects that are particularly promising for the treatment of various types of cancer. The bulk of studies carried out to date have focused on apricot extracts, amygdalin, and amygdalin-containing fractions using in vitro studies on different cancer cell lines, as well as in experimental animal models. More research is warranted to explore the biological effects of apricot extracts/constituents in clinical studies, as well as to investigate the nature of the compounds (or combination of compounds) that are responsible for these effects. It should be mentioned here that the use of laetrile, a synthetic analogue of amygdalin marketed for the treatment of cancer, is not advised, as this poisonous compound has shown little anticancer activity in vivo. The mechanisms involved in the anticancer activity of apricot are varied and include reducing cell proliferation, inducing autophagy, inducing apoptosis, protecting tissues/organs against oxidative damage, and reducing inflammation, angiogenesis, and telomerase activity. Some apricot extracts (and amygdalin) have been reported to selectively target acute leukemia and pancreatic cancer cells without any significant effect on normal cells. These are interesting observations resulting from in vitro experiments that should be explored further in animal models and clinical studies. Some reports have indicated that the selection of particular apricot cultivars could influence anticancer activity; therefore, this should be taken into account in future studies to obtain the strongest activity possible. Apricot preparations have also been shown to reduce some of the adverse effects associated with cancer treatments. In colon cancer cells, apricot extracts have been demonstrated to inhibit P-glycoprotein-mediated efflux mechanisms, suggesting their potential role as adjuvants to cancer treatments by reducing drug resistance.

Future perspectives should include translational pharmacological studies that accurately determine pharmacologically active doses in humans and potential side effects not yet reported. In addition, more pharmaceutical forms based on nanocarriers are needed for target transport in organs and to increase the bioavailability of bioactive compounds of *P. armeniaca* species. As well as investigating the potential of apricot extracts/constituents as complementary and alternative medicinal products to treat cancer, additional animal and clinical studies are required to evaluate the impact of the consumption of apricot fruits as part of a healthy diet on the prevention of carcinogenesis. It is hoped that this updated review will stimulate further investigations on the chemistry and biology of *P. armeniaca* L.

## Data Availability

Not applicable.
